# Biogenic Selenium Nanoparticles Synthesized Using Alginate Oligosaccharides Attenuate Heat Stress-Induced Impairment of Breast Meat Quality via Regulating Oxidative Stress, Metabolome and Ferroptosis in Broilers

**DOI:** 10.3390/antiox12122032

**Published:** 2023-11-22

**Authors:** Yu-Ying Yang, Yu-Chen An, Shu-Yue Zhang, Meng-Yi Huang, Xue-Qing Ye, Zhi-Hui Zhao, Wen-Chao Liu

**Affiliations:** 1 Department of Animal Science, College of Coastal Agricultural Sciences, Guangdong Ocean University, Zhanjiang 524088, China; yangyuying@stu.gdou.edu.cn (Y.-Y.Y.); zhangshuyue@stu.gdou.edu.cn (S.-Y.Z.); huangmengyi@stu.gdou.edu.cn (M.-Y.H.); yexueqing1@stu.gdou.edu.cn (X.-Q.Y.); 2 School of Computer Science and Engineering, Yangjiang Campus, Guangdong Ocean University, Yangjiang 529500, China; anyuchen@gdou.edu.cn

**Keywords:** antioxidant capacity, biogenic selenium nanoparticles, broilers, breast muscle, heat stress, meat quality

## Abstract

Selenium (Se) is an indispensable trace element with versatile functions in antioxidant defense in poultry. In our previous study, we synthesized a novel type of biogenic selenium nanoparticle based on alginate oligosaccharides (SeNPs-AOS), and found that the particles are sized around 80 nm with an 8% Se content, and the dietary addition of 5 mg/kg of SeNPs-AOS could effectively alleviate the deleterious effects of heat stress (HS) in broilers, but it is still unclear whether SeNPs-AOS can improve the meat quality. Therefore, the aim of this study was to evaluate the protective effects of SeNPs-AOS on breast meat quality in heat-stressed broilers, and explore the relevant mechanisms. Birds at the age of 21 days were randomly divided into four groups with six replicates per group (eight broilers per replicate) according to a 2 × 2 experimental design, using HS (33 ± 2 °C, 10 h/day vs. thermoneutral, TN, under 23 ± 1.5 °C) and SeNPs-AOS (5 mg/kg feed vs. no inclusion) as variables. The results showed that dietary SeNPs-AOS decreased the cooking loss (*p* < 0.05), freezing loss (*p* < 0.001), and shear force (*p* < 0.01) of breast muscle in heat-stressed broilers. The non-targeted metabolomics analysis of the breast muscle identified 78 differential metabolites between the HS and HS + SeNPs-AOS groups, mainly enriched in the arginine and proline metabolism, β-alanine metabolism, D-arginine and D-ornithine metabolism, pantothenate, and CoA biosynthesis pathways (*p* < 0.05). Meanwhile, supplementation with SeNPs-AOS increased the levels of the total antioxidant capacity (T-AOC), the activities of catalase (CAT) and glutathione peroxidase (GSH-Px), and decreased the content of malondialdehyde (MDA) in the breast muscle (*p* < 0.05) in broilers under HS exposure. Additionally, SeNPs-AOS upregulated the mRNA expression of *CAT*, *GPX1*, *GPX3*, *heme oxygenase-1* (*HO-1*), *masculoaponeurotic fibrosarcoma G* (*MafG)*, *MafK*, *selenoprotein W* (*SELENOW)*, *SELENOK*, *ferritin heavy polypeptide-1* (*FTH1*), *Ferroportin 1* (*Fpn1*), and *nuclear factor erythroid 2-related factor 2* (*Nrf2)* (*p* < 0.05), while it downregulated Kelch-like ECH-associated pro-36 tein 1 (*Keap1*) and prostaglandin-endoperoxide Synthase 2 (*PTGS2*) expression (*p* < 0.05) in broilers under HS. These findings demonstrated that the dietary addition of SeNPs-AOS mitigated HS-induced oxidative damage and metabolite changes in the breast muscle of broilers, which may be related to the regulation of the Nrf2 signaling pathway and selenoprotein synthesis. In addition, SeNPs-AOS upregulated the breast muscle gene expression of anti-ferroptosis-related molecules in broilers under HS, suggesting that SeNPs-AOS can be used as novel Se supplements against HS in broilers.

## 1. Introduction

Currently, in the context of global warming, heat stress (HS) has become a prime environmental stressor in broiler production [1,2]. HS not only adversely affects the growth and feed conversion rate of broilers [3] but also has a profound influence on muscle physiology and metabolism, ultimately impairing the chicken quality [4]. For instance, a lower pH, higher shear force, and freezing loss of the muscle were observed in broilers under HS [5,6]. Reactive oxygen species (ROS) accumulation is one of the main reasons for poorer meat quality in broilers subjected to HS. It has been confirmed that HS caused oxidative damage to muscle by generating excessive ROS, thus leading to the oxidation of muscle fatty acids and proteins, reduction of flavor, and the shortened shelf life of chicken [7]. Therefore, oxidative damage is considered to be the major factor of meat spoilage in broilers under HS, which is manifested by meat discoloration, a decrease in the water retention capacity, and the loss of nutritional value [8]. In addition, ferroptosis is usually accompanied by oxidative stress due to the phospholipid peroxidation of cell membranes [9]. Hence, the muscle cells are prone to ferroptosis under the oxidative conditions caused by HS, thereby exacerbating the impairment of muscle edible quality, which is a new framework of chicken quality reduction caused by thermal feeding conditions [10]. Notably, nutritional manipulation is an available option and has been used to minimize the HS-induced negative effects on the meat quality of broilers [11].

Selenium (Se) is an essential trace element for poultry and a component of selenomethionine, selenocysteine, and their oxidized forms, which are also substrates of various enzymes, including the antioxidant glutathione peroxidase (GPx) and other enzymes with antioxidant functions [12]. Meanwhile, Se acts as a co-factor for antioxidants and is involved in free radical scavenging, protects enzymes and nucleic acids from the deleterious impacts of ROS, and prevents cell membranes and organelles from lipid peroxidation, thereby greatly alleviating oxidative damage in heat-stressed broilers [13]. On the other hand, it has been demonstrated that dietary Se deficiency induces oxidative stress, inflammatory damage, and apoptosis in a variety of tissues [14], and adversely affects broiler performance [15]. As a new form of Se supplement, selenium nanoparticles (SeNPs) can maximize the beneficial effects of Se because of their high surface activity and nanoscale effect [16]. Compared with organic and inorganic selenium, SeNPs have lower toxicity and higher biological activity [17]. Studies have shown that the dietary inclusion of SeNPs could increase the content of polyunsaturated fatty acids (PUFAs) and protect lipids from ROS damage, and that SeNPs have better resistance to oxidative stress than inorganic and organic selenium in broilers [18]. However, SeNPs are unstable in storage and easily form precipitation, which influence their application in practice [19,20]. Conversely, biogenic SeNPs has been proven to be more stable in storage and more conducive to practical application compared to ordinary SeNPs [21], and dietary biogenic SeNPs have been reported to improve antioxidant capacity and meat quality in broilers [22,23].

Alginate oligosaccharides (AOSs) are naturally occurring anionic polymers isolated from brown seaweed, which have antioxidant and other biological activities [24]. We previously synthesized a new type of biogenic SeNP using AOS, and we speculated that the preparation of biogenic SeNPs using AOSs possesses both Se and AOS bioactivity simultaneously, and the efficacy of Se can be enhanced through nano-size effects [25]. However, the protective effects of SeNPs-AOS on breast meat quality in heat-stressed broilers and the mode of action are still unclear. Therefore, the present study aimed to evaluate whether SeNPs-AOS could improve the antioxidant capacity, metabolite composition, and ferroptosis to ameliorate the HS-induced impairment of breast meat quality in broilers.

## 2. Materials and Methods

### 2.1. Biosynthesis of SeNPs-AOS

The biosynthesis of SeNPs-AOS was achieved by reacting Na_2_SeO_3_ with Vc, while AOS served as a polymer template for the reaction. The reaction conditions, including reaction temperature, concentration of AOS, and reaction time, were selected through orthogonal experiments. It was found that the stable SeNPs-AOS products could be prepared under the conditions of AOS concentration of 400 mg/mL, reaction temperature of 60 °C, and reaction time of 30 min. Then, the SeNPs-AOS were characterized using scanning electron microscopy (SEM) and energy-dispersive spectroscopy (EDS), and demonstrated that the Se particle size was 80 nm and the Se content was 8%. In addition, supplementation with 5 mg/kg SeNPs-AOS had a significant anti-HS effect through an in vivo experiment. The specific preparation process, chemical components of AOS, and other details were described in our previous study [25].

### 2.2. Birds, Experimental Design, and Management

A total of 210 1-day-old unsexed Arbor Acres broilers (CP group, Zhanjiang, China) were raised to 21 days of age, from which 192 broilers with similar body weight were selected and divided into four groups according to a 2 × 2 trial design: thermoneutral group (TN), TN + SeNPs-AOS group, heat stress group (HS), and HS + SeNPs-AOS group. Each group had six replicates with eight chickens per replicate. The TN group and HS group were fed a basal diet (without antibiotics), and the additive group was supplemented with 5 mg/kg SeNPs-AOS in basal diet. The basal diet was formulated with reference to the NRC (1994), and the basal diet composition and nutritional levels are shown in Table S1. The Se content of the basal diet was 0.282 mg/kg, and the Se content of SeNPs-AOS added group was 0.696 mg/kg.

The feeding experimental period was 21 days, and the chickens were managed by free feeding and watering. The ambient temperature of the TN groups was maintained at 23 ± 1.5 °C; the ambient temperature of the HS group (8:00–18:00, 10 h/day) was maintained at 33 ± 2 °C, and ambient temperature was the same as that of the TN group during 18:00–8:00. The relative humidity was maintained at 60–75% for all groups.

### 2.3. Sample Collection

Samples of the breast muscle were collected on the 21st day of the experiment (42-days-old). From the replicate cages of each treatment group, one chicken weighing close to the average weight of the cage was randomly selected for slaughter, and the left breast muscle was collected for meat quality testing, while the right breast muscle was collected and stored in the refrigerator at −80 °C for the determination of metabolome, antioxidant capacity, and gene expression.

### 2.4. Measurement of Meat Quality

The breast muscle rate was measured according to the following formula: total breast muscle weight/live body weight. The pH of the breast muscle was determined by a pH meter after broiler slaughter; the brightness (L*), yellowness (b*), and redness (a*) values of the breast muscle were determined by a meat colorimeter within 2 h of slaughter. Determination of drip loss, freezing loss, and cooking loss was based on the report of Khajeh Bami et al. [26]. A regularly shaped 3 cm (length) × 2 cm (width) × 1 cm (thickness) meat sample was taken, weighed (W1), and then hung along the direction of the muscle fibers in a refrigerator at 4 °C. After 24 h, it was taken out to absorb the surface water with filter paper and weighed (W2), and the drip loss = (W1 − W2)/W1. Within 2 h of slaughter sampling, 5 g of muscle sample (W1) was placed in a −20 °C refrigerator for 24 h, and thawed under room temperature conditions and then the surface water was absorbed with filter paper and weighed (W2); freezing loss = (W1 − W2)/W1. After slaughter, about 15 g of regular shaped meat samples were cut and weighed after removing the fascia (W1), heated in a water bath at 80 °C for exactly 30 min, then the meat samples were taken out and cooled statically to room temperature, and weighed after drying the surface water with filter paper (W2); cooking loss = (W1 − W2)/W1. After determination of cooking loss, regularly shaped 3 cm (length) × 1 cm (width) × 1 cm (thickness) meat samples were cut from each sample, and the shear force was determined using the method of Chen et al. [6].

### 2.5. Non-Targeted Metabolomics Analysis

A 2 g breast muscle sample was collected from each slaughtered broiler, which were sent to Suzhou PANOMIX Biomedical Technology Co., Ltd. (Suzhou, China) for the determination of non-targeted metabolomics. The samples were processed and subjected to liquid chromatography (LC)-mass spectrum (MS) detection. The assay methods and data analysis are referenced to Yue et al. [27]. Briefly, the breast samples were first used for metabolite extraction, and then LC-MS determination was performed. The LC analysis was conducted using Vanquish UHPLC System (Thermo Fisher Scientific, Waltham, MA, USA). MS detection of metabolites was carried out using Orbitrap Exploris 120 (Thermo Fisher Scientific, Waltham, MA, USA). The raw data were firstly converted to mzXML format using MSConvert in ProteoWizard software package (La Jolla, CA, USA, v3.0.8789). The Ropls software (Auckland, New Zealand, v2.1) was used for multivariate data analyses and modeling. Differential metabolites were subjected to pathway enrichment analysis using MetaboAnalyst software (Edmonton, AB, Canada, v3.0).

### 2.6. Antioxidant Capacity Analysis

Samples from breast muscle were prepared in the ratio of 1:9 weight to saline (concentration 0.9%) by volume, and then crushed into 10% tissue homogenate using KZ-II tissue homogenizer from Wuhan Sevier Biotechnology Co., Ltd. (Wuhan, China). The protein concentration of the samples was determined using TAKARA Protein Concentration Assay Kit (Osaka, Japan, Item No. T9300 A), and then the antioxidant kits from Nanjing Jiancheng Bioengineering Institute (Nanjing, China) were used to determine the total antioxidant capacity (T-AOC) (Item No. A015-2-1), catalase (CAT) (Item No. A007-1-1), glutathione peroxidase (GSH-Px) (Item No. A005-1-2), total superoxide dismutase (T-SOD) (Item No. A001-1-1), malondialdehyde (MDA) (Item No. A003-1-2), and glutathione transferase (GST) (Item No. A004-1-1) indexes of the samples.

### 2.7. Determination of Antioxidant, Selenoprotein, and Ferroptosis-Related Gene Expression

Total RNA from breast muscle was extracted according to the TRIzol total RNA extraction reagents and instructions. RNA was reverse transcribed into cDNA using the reverse transcription kit HiScript II Q RT SuperMix for qPCR from Nanjing Vazyme Bioscience and Technology Company Limited, Nanjing, China (Item No. R223-01), ChamQ Universal SYBR qPCR Master Mix (Item No. Q711-02) was used for qPCR experiments, qPCR reaction system was Mix (10 μL), H_2_O (8.2 μL), cDNA (1 μL), and F/R (0.4 μL/0.4 μL), and the information on the primers is presented in Table S2. Specific sequences of the related genes were quantified by qPCR, and the relative mRNA expression of the related genes was calculated by the 2^−ΔΔCT^ method, the *β-actin* was used as the internal reference gene.

### 2.8. Statistical Analysis

All data except metabolomics were analyzed using two-way analysis of variance (ANOVA) of general linear modelling (GLM) procedure in SAS software (v9.4), and Tukey’s test was used to compare the significance of differences between groups. Metabolomic data analysis was described in Section 2.5. A value of 0.05 ≤ *p* < 0.10 indicates that the difference tends to be significant, *p* < 0.05 indicates that the difference is significant, and *p* < 0.01 indicates that the difference is highly significant.

## 3. Results

### 3.1. Meat Quality

As shown in Table 1, the shear force (*p* < 0.01), cooking loss (*p* < 0.01), and freezing loss (*p* < 0.05) were elevated, and the pH (0 min) (*p* < 0.05) was reduced in the HS group, the dietary SeNPs-AOS decreased the freezing loss (*p* < 0.01) and shear force (*p* < 0.01) of the breast muscle in the broilers under HS. There was an interactive effect of temperature and SeNPs-AOS on the cooking loss, freezing loss, and shear force of the breast muscle (*p* < 0.01).

### 3.2. Non-Targeted Metabolomics

#### 3.2.1. Multivariate Statistical Analysis

**Figure 1:**
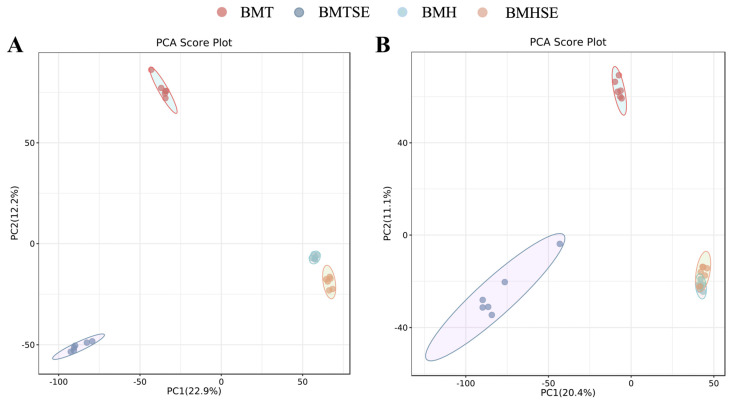

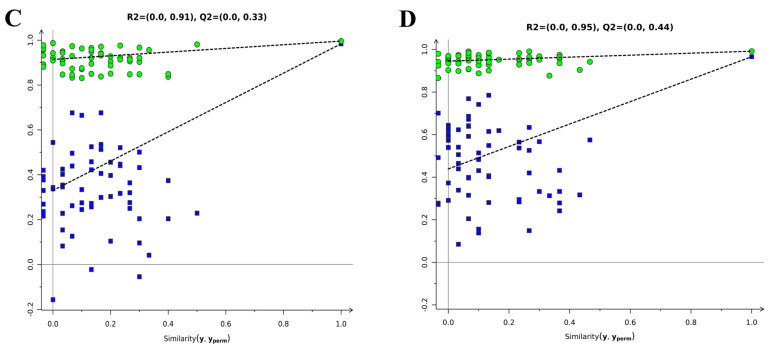
Multivariate statistical analysis between groups. BMT, thermoneutral group; BMTSE, thermoneutral SeNPs-AOS-added group; BMH, heat stress control group; BMHSE, heat stress SeNPs-AOS-added group; (**A**) principal component analysis (PCA) score plot-based positive ion mode results; (**B**) PCA score plot-based negative ion mode results; (**C**) PLS-DA substitution test plot based on the positive ion model; (**D**) PLS-DA substitution test plot based on the negative ion model. In Figure 1C,D, green dots represent R2 (the proportion of data variance or variation that the current model can explain), blue dots represent Q2 (the proportion of data variance that the current model can predict), dashed lines represent regression analysis lines, and solid lines represent horizontal and vertical coordinates starting from 0.

Based on the positive and negative ion patterns, all the QC samples were densely distributed on the PCA analysis plot, indicating good reproducibility (Figure 1A,B), while all the blue Q2 points in the PLS-DA (R2Y = 0.91, Q2Y = 0.33) plot were lower than the original blue Q2 points on the far right, suggesting that the results were reliable and valid (Figure 1C,D).

#### 3.2.2. Differential Metabolites Identification

**Figure 2:**
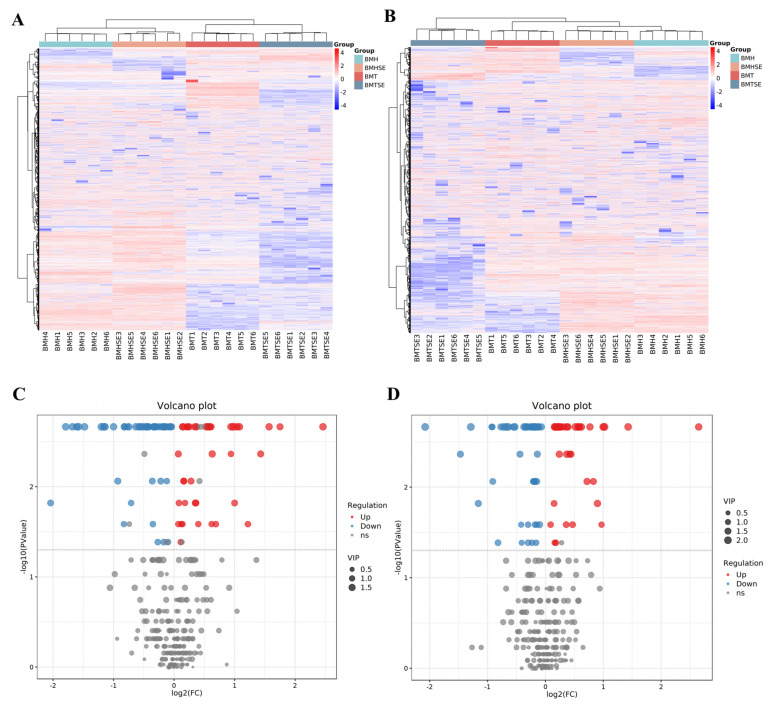
Analysis and identification of differential metabolites in the breast muscle between groups. BMT, thermoneutral group; BMTSE, thermoneutral SeNPs-AOS-added group; BMH, heat stress control group; BMHSE, heat stress SeNPs-AOS-added group; (**A**) heatmap clustering of different groups in positive ion mode; (**B**) heatmap clustering of different groups in negative ion mode; (**C**) volcano map of differential metabolites in normothermic thermoneutral and heat-stressed groups; (**D**) volcano map of differential metabolites in heat stress and heat stress control groups.

The metabolite difference between the groups was analyzed by heatmaps and a volcano diagram, and a total of 312 metabolites were identified in both the positive and negative ion modes (Figure 2A,B). A total of 84 differential metabolites were detected between the TN and HS group; 78 differential metabolites were found between the HS and HS + SeNPs-AOS group (Figure 2C,D). These differential metabolites mainly include fatty acids, organic acids, and amino acids. Compared with the TN group, 39 metabolites were upregulated and 45 metabolites were downregulated in the HS group, including elevated cholesterol and decreased L-histidine, L-glutamine, and L-malic acid (Table S3). Compared to the HS group, there were 35 metabolites elevated and 43 metabolites reduced in the HS + SeNPs-AOS group, including the elevated L-malate, L-glutamate, and inosine (Table S4).

#### 3.2.3. Metabolic Pathways of Differential Metabolites

**Figure 3:**
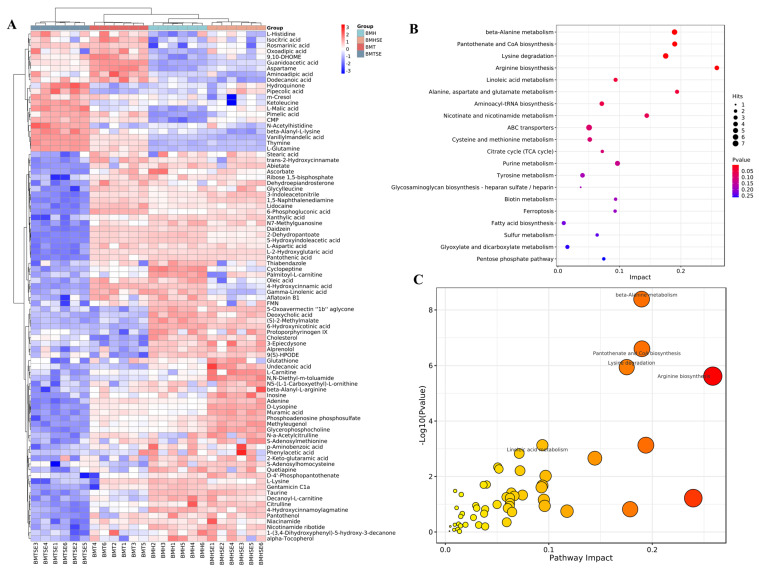
Intergroup enrichment analysis of differential metabolites and metabolic pathways. BMT, thermoneutral group; BMTSE, thermoneutral SeNPs-AOS-added group; BMH, heat stress control group; BMHSE, heat stress SeNPs-AOS-added group; (**A**) heatmap of differential metabolites; (**B**) pathway prediction of differential metabolites based on KEGG analysis; (**C**) pathway enrichment of differential metabolites, the color of the dots is related to the *p*-value, with darker colors indicating smaller *p*-values and lighter colors indicating larger *p*-values; the size of the dots represents the impact value, and a larger impact value indicates a larger dot.

In order to explore the metabolic pathways of the differential metabolites between groups, an enrichment analysis of 89 differential metabolites were performed (Figure 3A). Among the 20 KEGG pathways, six metabolic pathways were significantly enriched (*p* < 0.05), including β-alanine metabolism, pantothenic acid and CoA biosynthesis, lysine degradation, arginine biosynthesis, linoleic acid metabolism, as well as alanine, aspartate, and glutamate metabolism (Figure 3B,C).

### 3.3. Antioxidant Capacity

As shown in Table 2, HS decreased the level of T-AOC, reduced the activities of the T-SOD, CAT, and GSH-Px enzymes, and increased the content of MDA (*p* < 0.01). Dietary SeNPs-AOS elevated the activities of the CAT, GST, and GSH-Px enzymes, and decreased the MDA content in the breast muscle of the broilers under HS (*p* < 0.05). There was an interaction between temperature and SeNPs-AOS on the T-AOC, CAT, MDA, and GSH-Px (*p* < 0.05).

### 3.4. Determination of Antioxidant, Selenoprotein, and Ferroptosis-Related Gene Expression

**Figure 4:**
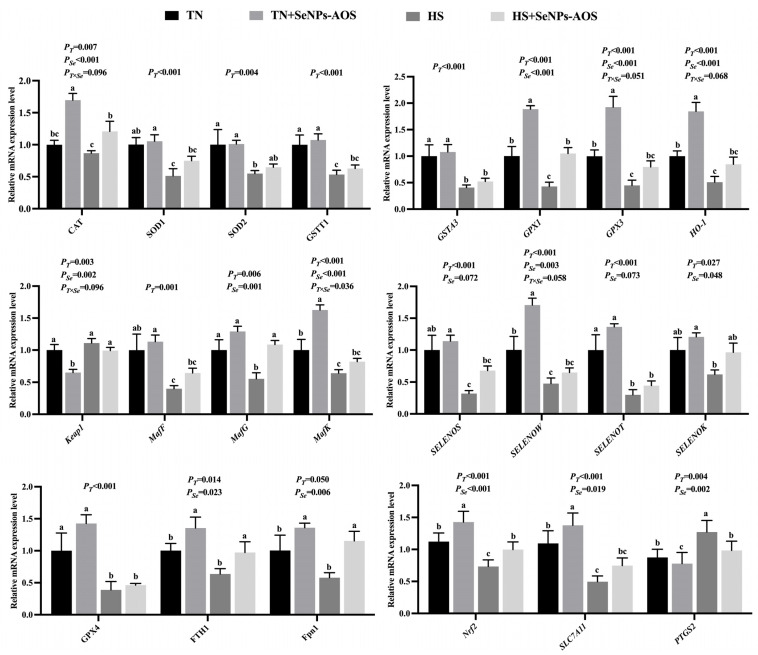
Effect of SeNPs-AOS on mRNA expression of breast muscle-related genes in heat-stressed broilers. TN, thermoneutral; TN + SeNPs-AOS, basal diet + SeNPs-AOS (5 mg/kg); HS, heat stress control; HS+ SeNPs-AOS, basal diet + SeNPs-AOS; *P_T_*, ambient temperature main effect *p* value; *P_Se_*, SeNPs-AOS main effect *p* value; *P_T×Se_*, *p* value for the interaction effect between ambient temperature and SeNPs-AOS. ^a,b,c^ Different superscript letters suggest a significant difference between groups; 0.05 ≤ *p* < 0.10 indicates that the difference tends to be significant, *p* < 0.05 indicates that the difference is significant, *p* < 0.01 indicates that the difference is highly significant.

As presented in Figure 4, HS reduced the mRNA expression of *CAT*, *SOD1*, *SOD2*, *GSTT1*, *GSTA3*, *GPX1*, *GPX3*, heme oxygenase-1 (*HO-1*), masculoaponeurotic fibrosarcoma F (*MafF*), *MafG*, *MafK*, selenoprotein S (*SELENOS*), *SWLENOW*, *SELENOT*, *SELENOK*, *GPX4*, ferritin heavy polypeptide-1 (*FTH1*), nuclear factor (erythroid-derived-2)-like 2 (*Nrf2*), and solute carrier family 7 member 11 (*SLC7A11*) (*p* < 0.05), and elevated the mRNA expression of Kelch-like ECH-associated protein 1 (*Keap*1) and post-transcriptional gene silencing-2 (*PTGS2*) (*p* < 0.01). The supplementation with SeNPs-AOS upregulated the mRNA expression of *CAT*, *GPX1*, *GPX3*, *HO-1*, *MafG*, *MafK*, *SELENOW*, *SELENOK*, *FTH1*, *Fpn1*, *Nrf2*, and *SLC7A11* (*p* < 0.05), and downregulated the mRNA expression of *Keap*1 and *PTGS2* of the breast muscle in the broilers subjected to HS (*p* < 0.01). There was an interaction between temperature and SeNPs-AOS on the mRNA expression of the *MafK* gene (*p* < 0.05).

## 4. Discussion

Chicken has become the main animal source food for humans worldwide. Nevertheless, global warming makes broilers susceptible to HS and results in a decrease in chicken quality [28]. HS conditions adversely affect glycolytic metabolism, protein synthesis, and fat deposition in broilers, and simultaneously over-produces reactive oxygen species (ROS), thereby impairing the meat quality [29]. Extensive studies have confirmed the deleterious influences of HS on the chicken quality in terms of increased drip loss, cooking loss, and muscle brightness, and reduced pH and redness [30,31,32]. Additionally, HS reduces feed intake, resulting in a shortage of myoglobin and other proteins that regulate meat color, which in turn induces an increase in meat brightness and a lower degree of redness [33]. After slaughter, a large amount of lactic acid from glycolysis accumulates in the muscle, which reduces muscle pH, leading to protein denaturation and ultimately reducing the muscle tethering capacity [34]. Furthermore, free radicals and lipid peroxides produced by HS cause a decrease in antioxidant activity in the muscle, thereby destroying the structure and function of the biofilm and leading to the water loss and atrophy of muscle cells [35]. In this study, HS boosted the breast shear force, cooking loss, and freezing loss, while it reduced the breast redness and pH (0 min) values, which are consistent with previous studies [36,37,38,39]. It is interesting that dietary SeNPs-AOS reduced the shear force and freezing loss of breast muscle in the heat-stressed broilers. Similarly, Mohamedde et al. [40] indicated that supplementation with inorganic Se and bacterial selenoproteins reduced the drip loss, cooking loss, and shear force of breast muscle. This may be associated with the respective antioxidant functions of Se and AOS in SeNPs-AOS. Also, the biogenic SeNPs could enhance Se bioactivity through nanoscale effects to promote the antioxidant properties [41]. Se is a key component of Se-containing proteins and enzymes, such as GSH-Px [42,43], which play a crucial role in the antioxidant defense system [44]. It has been reported that dietary Se enhanced the GPx activity of muscle, which contributed to the improvement of meat quality [45]. Zhou et al. [46] suggested that supplementation with SeNPs was effective in upgrading the Se content of the tissues as well as the meat quality. In addition, in AOS as the degradation products of alginate, the antioxidant activity has been demonstrated [47]. Hence, it is reasonable to speculate that SeNPs-AOS reduced the adverse effects of HS on meat quality and may be partial via improving the antioxidant capacity of breast muscle.

Metabolites are another important indicator of meat quality as they can characterize muscle composition and metabolic status [48]. Metabolomics analysis is a new technique for detecting changes in endogenous metabolites affected by external stimulated or internal disturbances, which enables the diagnosis and prediction of metabolite change [49]. LC-MS, as one of the assays to detect metabolites, is able to provide a comprehensive coverage of substances and is considered to be a suitable technique for studying metabolism [50]. Numerous studies have documented that HS reduced meat quality by altering energy metabolism, amino acid transport, glycolysis, intramuscular fat deposition, and protein synthesis [36,51,52,53]. In our study, a total of 312 metabolites were identified based on metabolomics analysis, mainly including organic acids, sugars, and amino acids, which were enriched in amino acid and organic acid metabolism using the KEGG analysis. The content of free amino acids, unsaturated fatty acids, and nucleotides (inosine-5′-monophosphate [IMP], adenosine-5′-monophosphate [AMP], and inosine) has been reported as detection indicators for meat flavor [54]. Amino acids (glutamic acid, aspartic acid, glycine, arginine, etc.) are not only essential for proteins, but also influence the synthesis of other muscle components, which are important for the specific flavor of meat [55]. Meanwhile, amino acids and their derivatives can increase the concentration of free amino acids, enhance the antioxidant function and immunity, and indirectly affect meat quality [56]. Broilers under HS conditions have altered amino acid metabolism and accelerated protein breakdown, thus decreasing protein deposition and ultimately impairing the meat quality [57]. On the other hand, HS adversely affects lipid metabolism and reduces the lipolytic capacity of animals, leading to metabolic disorders as well as fat deposition [58]. In this study, the glutamine, histidine, and L-malate were decreased and the cholesterol were increased by HS exposure. Glutamate and inosine are the taste-active components of chicken and there is a synergistic effect between them [59]. L-glutamic acid is closely related to the meat quality (e.g., shear force, cooking loss, drip loss) and when L-glutamic acid is elevated, the shear force, cooking loss, and drip loss are decreased. It is worth mentioning that the present findings demonstrated that dietary SeNPs-AOS elevated the L-glutamate and inosine levels of breast muscle in heat-stressed broilers. This is in agreement with Tian et al. [60], who showed that supplementation with yeast Se increased the concentration of glutamate and flavor amino acids in the meat, which could contribute to the enhancement of meat quality. Therefore, the regulation of amino acid metabolism is one of the reasons why dietary SeNPs-AOS alleviate HS-induced breast muscle quality damage.

HS generates large amounts of ROS, and when the balance between the production and elimination of ROS is disrupted, the body’s antioxidant level decreases, which in turn induces cellular oxidative stress [61]. The present study indicated that HS decreased the level of T-AOC, reduced the activity of the T-SOD, CAT, and GSH-Px enzymes, and increased the content of MDA. Antioxidant enzymes are important factors for maintaining redox balance, and the scavenging of free radicals in the muscle depends on a variety of antioxidant enzymes, including SOD, CAT, and GSH-Px [62]. MDA, as a lipid peroxide, is widely recognized as a biomarker of oxidative stress [63]. Supplementation with Se has been reported to enhance the antioxidant capacity in the muscle and other organs [64]. Consistently, we found that dietary SeNPs-AOS increased the levels of T-AOC, elevated CAT and GSH-Px activities, and decreased the MDA content, thus easing the oxidative damage of breast muscle caused by HS. Hence, the findings of antioxidant performance confirmed that the beneficial effect of SeNPs-AOS on meat quality could be attributed to the improvement of the peroxidation state in the breast muscle.

Selenium acts as an essential trace element in animals and plays a key role in various biological functions, mainly in the form of selenoproteins, which exhibit wide-ranging physiological functions in the body [65]. Twenty-four selenoproteins have been identified in broilers (SELENOK, SELENOS, SELENOT, etc.). These proteins are involved in the maintenance of redox homeostasis [66]. In the present study, HS reduced the mRNA expression of selenoproteins such as *GPx1*, *GPx3*, *SELENOS*, *SELENOW*, etc. This is similar to the results of Cao et al. [67], who demonstrated that HS downregulated the expression of *GPx2*, *GPx6*, *Txnrd*, *Selh*, *Selm*, and *Selx* in IPEG-J2 cells. However, our research found that dietary supplementation with SeNPs-AOS upregulated the mRNA expression of selenoprotein genes including *GPx3*, *SELENOW*, and *SELENOK*. In agreement, a previous study suggested that dietary selenite promoted the mRNA expression of *GPx1*, *SELENOW*, and *SELENOP* in an animal model [68], indicating that supplementation with SeNPs-AOS ameliorate oxidative damage also through promoting selenoprotein biosynthesis. The Nrf2/Keap1 is a key pathway of the oxidative stress response. In the physiological condition, Nrf2 binds to Keap1 in the cytoplasm, but when the cell undergoes oxidative stress, Nrf2 disassembles from Keap1 and translocates to the nucleus, then activates the expression of a series of downstream antioxidant genes (including selenoprotein coding genes) by binding to antioxidant elements (AREs) to enhance the antioxidant capacity of the cell [69]. In this experiment, HS downregulated the mRNA relative expression of *CAT*, *SOD1*, *GSTA3*, *MafF*, *Nrf2* and other genes, and upregulated the mRNA expression of *Keap1*. Notably, dietary SeNPs-AOS upregulated the mRNA expression of *Nrf2* and the downstream antioxidant related genes. Similar to our study, a previous report demonstrated that dietary biogenic SeNPs were able to protect the mouse intestinal barrier function from oxidative stress by activating Nrf2 signaling and its downstream genes [70]. Additionally, it should be noted that the AOS could also reduce the oxidative damage through activating the Nrf2 pathway [71]. Therefore, Se and AOS may both contribute to SeNPs-AOS in alleviating breast oxidative stress by targeting Nrf2 signaling, and, unlike the traditional forms of selenium, its unique nanoscale effect may enhance such beneficial regulatory roles.

Ferroptosis is a novel form of cell death, mainly caused by the accumulation of excessive iron-dependent lipid peroxidation, which is also an important adverse consequence of oxidative stress and can exacerbate the impairment of meat quality [72]. Nrf2 is not only a key regulator of cellular antioxidant, but also can inhibit ferroptosis by modulating the expression of genes involved in iron metabolism (e.g., *FTH1* and *Fpn1*) and glutathione synthesis and metabolism (e.g., *GPX4* and *SLC7A11*) [73]. The results of this experiment showed that HS reduced the expression of *GPX4* and *SLC7A11*, and increased the expression of the *PTGS2* gene. Conversely, supplementation with SeNPs-AOS raised the levels of *FTH1*, *Fpn1*, and lowered the levels of *PTGS2*. Iron transport mechanisms are capable of maintaining intracellular iron homeostasis [74]. Under normal physiological conditions, almost all iron in the body is tightly bound to transferrin and ferritin [75]. FTH1 is a negative regulator of ferroptosis [76] and protects cells from ferroptosis by rapidly converting Fe^2+^ to Fe^3+^ and storing iron in ferritin [77]. As an iron transporter, Fpn1 can alleviate ferroptosis by reducing the overload of Fe^2+^ in the cell [78]. PTGS2 is a biomarker of ferroptosis [79] and plays a role as a pivotal gene in the biology of ferroptosis [80], and the downregulation of the *PTGS2* expression level suggests the alleviation of ferroptosis. Previously, Zhao et al. [81] have shown that supplementation with Se reduced ferroptosis in heart cells. It has been found that Se supplementation could activate the Nrf2/transferrin signaling pathway to suppress ferroptosis [82,83], suggesting that SeNPs-AOS could inhibit oxidative stress by regulating the Nrf2 pathway, which might also suppress ferroptosis through targeting Nrf2/transferrin pathway in the breast muscle of heat-stressed broilers. However, a Prussian blue staining and intracellular iron accumulation assay for the breast muscle were not performed in this study, so the anti-ferroptosis effects of SeNPs-AOS and the mechanism of action still need further confirmation.

## 5. Conclusions

**Figure 5:**
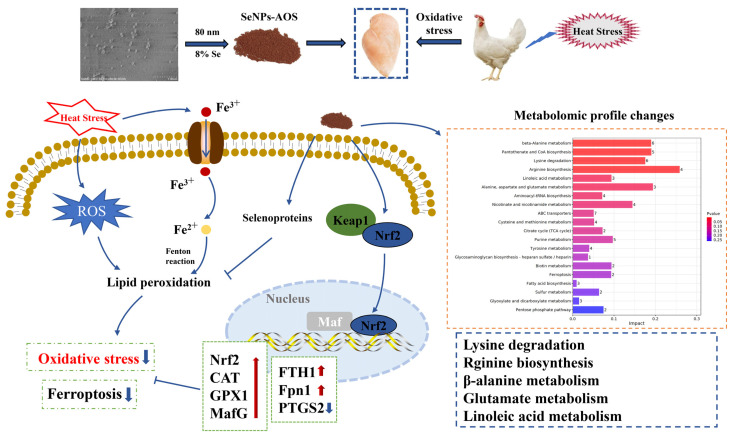
Proposed mechanism for SeNPs-AOS (biogenic selenium nanoparticles synthesized using alginate oligosaccharides) to attenuate heat stress-induced impairment of breast meat quality in broilers. The upward red arrow indicates the promoting effect of SeNPs-AOS, while the downward blue arrow indicates the reducing effect of SeNPs-AOS.

Collectively, we prepared biogenic SeNPs-AOS that could ameliorate HS-induced meat quality loss through reducing oxidative stress in the breast muscle of broilers. The beneficial effect may be associated with the activation of the Nrf2 signaling pathway and the promotion of selenoprotein synthesis. Additionally, SeNPs-AOS improved the breast meat quality in relation to the regulation of metabolites (especially the amino acid metabolism), and SeNPs-AOS could also upregulate the expression of the Nrf2/transferrin pathway-associated anti-ferroptosis genes of the breast muscle in broilers under HS (Figure 5). The current findings have provided novel insights into SeNPs-AOS, which serve as a new type of antioxidant to improve the meat quality in broilers raised in summer and/or tropical areas.

## Figures and Tables

**Table 1:** Effect of SeNPs-AOS on breast meat quality of heat-stressed broilers.

Items	TN		HS		SEM	*p*-Value		
	CON	SeNPs-AOS	CON	SeNPs-AOS		Temp.	SeNPs-AOS	Temp. × SeNPs-AOS
Breast muscle rate	0.25	0.25	0.24	0.25	0.009	0.516	0.986	0.870
pH (0 min)	6.77 ^a^	6.69 ^ab^	6.55 ^b^	6.63 ^ab^	0.055	0.022	0.952	0.145
pH (24 h)	6.49 ^a^	6.32 ^b^	6.38 ^ab^	6.34 ^ab^	0.051	0.400	0.063	0.214
Shear force	5.94 ^b^	5.91 ^b^	8.24 ^a^	5.88 ^b^	0.360	0.180	0.001	0.016
Drip loss	0.05	0.05	0.06	0.05	0.006	0.005	0.004	0.004
Cooking loss	0.23 ^b^	0.29 ^a^	0.31 ^a^	0.29 ^a^	0.010	0.002	0.048	0.001
Freezing loss	0.07 ^b^	0.06 ^bc^	0.09 ^a^	0.06 ^c^	0.003	0.045	<0.001	0.001
Lightness L*	47.65 ^a^	46.45 ^ab^	46.11 ^ab^	43.70 ^b^	1.071	0.059	0.106	0.579
Redness a*	3.30 ^ab^	3.47 ^a^	2.44 ^bc^	2.12 ^c^	0.293	0.001	0.792	0.413
Yellowness b*	12.23 ^ab^	13.88 ^a^	12.70 ^ab^	11.59 ^a^	0.685	0.197	0.698	0.056

SeNPs-AOS, biogenic selenium nanoparticles synthesized from alginate oligosaccharides; TN group, thermoneutral zone; HS group, heat stress; CON group, control group, basal diet without addition of SeNPs-AOS; SeNPs-AOS group, basal diet with addition of 5 mg/kg SeNPs-AOS; data are expressed as mean (6 replicates per treatment group, n = 6); SEM, standard error; temp., temperature; ^a,b,c^ different superscript letters suggest a significant difference between groups; 0.05 ≤ *p* < 0.10 indicates that the difference tends to be significant, *p* < 0.05 indicates that the difference is significant, *p* < 0.01 indicates that the difference is highly significant.

**Table 2:** Effect of SeNPs-AOS on antioxidant capacity of heat-stressed broiler breast muscle.

Items	TN		HS		SEM	*p*-Value		
	CON	SeNPs-AOS	CON	SeNPs-AOS		Temp.	SeNPs-AOS	Temp. × SeNPs-AOS
T-AOC, mmol/mg prot	0.29 ^a^	0.25 ^ab^	0.24 ^c^	0.23 ^bc^	19.747	0.002	0.804	0.037
T-SOD, U/mg prot	239.96 ^a^	231.04 ^a^	172.42 ^b^	186.94 ^b^	13.039	0.001	0.832	0.380
CAT, U/mg prot	5.20 ^a^	5.05 ^a^	2.10 ^b^	4.02 ^a^	0.404	<0.001	0.041	0.018
MDA, nmol/mg prot	0.39 ^b^	0.40 ^b^	0.66 ^a^	0.48 ^b^	0.037	0.001	0.030	0.015
GSH-Px, U/mg prot	25.96 ^a^	29.54 ^a^	11.23 ^b^	26.02 ^a^	1.804	<0.001	<0.001	0.006
GST, U/mg prot	29.26 ^ab^	35.55 ^a^	20.74 ^b^	30.43 ^ab^	3.428	0.06	0.030	0.625

SeNPs-AOS, biogenic selenium nanoparticles synthesized from alginate oligosaccharides; TN group, thermoneutral zone; HS group, heat stress; CON group, control group, basal diet without addition of SeNPs-AOS; SeNPs-AOS group, basal diet with addition of 5 mg/kg SeNPs-AOS; T-AOC, total antioxidant capacity; T-SOD, total superoxide dismutase; CAT, catalase; MDA, malondialdehyde; GSH-Px, glutathione peroxidase; GST, glutathione-S-transferase; temp., temperature; data are expressed as mean (6 replicates per treatment group, n = 6); SEM, standard error; ^a,b,c^ different superscript letters suggest a significant difference between groups; 0.05 ≤ *p* < 0.10 indicates that the difference tends to be significant, *p* < 0.05 indicates that the difference is significant, *p* < 0.01 indicates that the difference is highly significant.

## Data Availability

The data are contained within the article and Supplementary Materials.

## Supplementary Materials

The following supporting information can be downloaded at https://www.mdpi.com/article/10.3390/antiox12122032/s1: Table S1: Basal diet composition and ingredients (22–42 d); Table S2: Primers used in this study for quantitative real-time PCR; Table S3: Effect of heat stress on various differential metabolites in the breast muscle of broilers; Table S4: Effect of biogenic selenium nanoparticles synthesized from alginate oligosaccharides (SeNPs-AOS) on various differential metabolites in the breast muscle of broilers under heat stress.

## Abbreviations

AOS, alginate oligosaccharides; ANOVA, two-way analysis of variance; a*, redness; AMP, adenosine-5′-monophosphate; b*, yellowness; CAT, catalase; EDS, energy-dispersive spectroscopy; FTH1, ferritin heavy polypeptide-1; *Fpn1*, ferroportin 1; GSH-Px, glutathione peroxidase; GST, glutathione transferase; GLM, general linear modelling; HS, heat stress; *HO-1*, heme oxygenase-1; IMP, inosine-5′-monophosphate; *Keap1*, Kelch-like ECH-associated pro-36 tein 1; L*, brightness; MDA, malondialdehyde; *MafG*, masculoaponeurotic fibrosarcoma G; *MafF*, masculoaponeurotic fibrosarcoma F; *MafK*, masculoaponeurotic fibrosarcoma K; *Nrf2*, nuclear factor erythroid 2-related factor 2; Na_2_SeO_3_, sodium selenite; *PTGS2*, prostaglandin-endoperoxide synthase 2; PUFA, polyunsaturated fatty acid; ROS, reactive oxygen species; SeNPs, selenium nanoparticles; Se, selenium; SeNPs-AOS, biogenic selenium nanoparticles based on alginate oligosaccharides; *SELENOW*, selenoprotein *W*; *SELENOS*, selenoprotein S; *SELENOK*, selenoprotein K; *SELENOT*, selenoprotein T; SEM, scanning electron microscope; TN, thermoneutral zone; T-AOC, total antioxidant capacity; T-SOD, total superoxide dismutase; Vc, ascorbic acid.

## References

[1] Rostagno M.H. (2020). Effects of heat stress on the gut health of poultry. J. Anim. Sci..

[2] Song D.J., King A.J. (2015). Effects of heat stress on broiler meat quality. World’s Poult. Sci. J..

[3] Ebrahim R., Liang J.B., Jahromi M.F., Shokryazdan P., Ebrahimi M., Chen W.L., Goh Y.M. (2015). Effects of tannic acid on performance and fatty acid composition of breast muscle in broiler chickens under heat stress. Ital. J. Anim. Sci..

[4] Lu Z., He X.F., Ma B.B., Zhang L., Li J.L., Jiang Y., Zhou G.H., Gao F. (2017). Chronic heat stress impairs the quality of breast-muscle meat in broilers by affecting redox status and energy-substance metabolism. J. Agric. Food Chem..

[5] Pardo Z., Lara L., Nieto R., Fernández-Fígares I., Seiquer I. (2023). Muscle quality traits and oxidative status of Iberian pigs supplemented with zinc and betaine under heat stress. Meat Sci..

[6] Chen Y.P., Cheng Y.F., Du M.F., Zhou Y.M. (2021). Protective effects of dietary synbiotic supplementation on meat quality and oxidative status in broilers under heat stress. Environ. Sci. Pollut. Res..

[7] Song Z.H., Cheng K., Zheng X.C., Ahmad H., Zhang L.L., Wang T. (2018). Effects of dietary supplementation with enzymatically treated Artemisia annua on growth performance, intestinal morphology, digestive enzyme activities, immunity, and antioxidant capacity of heat-stressed broilers. Poult. Sci..

[8] Chen Z., Xing T., Li J., Zhang L., Jiang Y., Gao F. (2022). Oxidative stress impairs the meat quality of broiler by damaging mitochondrial function, affecting calcium metabolism and leading to ferroptosis. Anim. Biosci..

[9] Chen Y., Guo X., Zeng Y., Mo X., Hong S., He H., Li J., Fatima S., Liu Q. (2023). Oxidative stress induces mitochondrial iron overload and ferroptotic cell death. Sci. Rep..

[10] Chen G.H., Song C.C., Pantopoulos K., Wei X.L., Zheng H., Luo Z. (2022). Mitochondrial oxidative stress mediated Fe-induced ferroptosis via the NRF2-ARE pathway. Free Radic. Biol. Med..

[11] Zhang M., Dunshea F.R., Warner R.D., DiGiacomo K., Osei-Amponsah R., Chauhan S.S. (2020). Impacts of heat stress on meat quality and strategies for amelioration: A review. Int. J. Biometeorol..

[12] Gu Y., Qiu Y., Wei X., Li Z., Hu Z., Gu Y., Zhao Y., Wang Y., Yue T., Yuan Y. (2020). Characterization of selenium-containing polysaccharides isolated from selenium-enriched tea and its bioactivities. Food Chem..

[13] Bhattacharjee A., Basu A., Bhattacharya S. (2019). Selenium nanoparticles are less toxic than inorganic and organic selenium to mice in vivo. Nucleus.

[14] Zhang Z., Liu M., Guan Z., Yang J., Liu Z., Xu S. (2017). Disbalance of calcium regulation-related genes in broiler hearts induced by selenium deficiency. Avian Pathol..

[15] Bakhshalinejad R., Hassanabadi A., Swick R.A. (2019). Dietary sources and levels of selenium supplements affect growth performance, carcass yield, meat quality and tissue selenium deposition in broilers. Anim. Nutr..

[16] Xiao D., Li T., Huang X., Zhu K., Li Z., Dong Y., Wang L., Huang J. (2023). Advances in the Study of Selenium-Enriched Probiotics: From the Inorganic Se into Se Nanoparticles. Mol. Nutr. Food Res..

[17] Saffari S., Keyvanshokooh S., Zakeri M., Johari S., Pasha-Zanoosi H. (2017). Effects of different dietary selenium sources (sodium selenite, selenomethionine and nanoselenium) on growth performance, muscle composition, blood enzymes and antioxidant status of common carp (*Cyprinus carpio*). Aquac. Nutr..

[18] Bień D., Michalczuk M., Łysek-Gładysińska M., Jóźwik A., Wieczorek A., Matuszewski A., Kinsner M., Konieczka P. (2023). Nano-Sized Selenium Maintains Performance and Improves Health Status and Antioxidant Potential While Not Compromising Ultrastructure of Breast Muscle and Liver in Chickens. Antioxidants.

[19] Xu X., Pan Y., Liu X., Han Z., Chen S. (2023). Constructing Selenium Nanoparticles with Enhanced Storage Stability and Antioxidant Activities via Conformational Transition of Curdlan. Foods.

[20] Ji H., Lou X., Jiao J., Li Y., Dai K., Jia X. (2023). Preliminary Structural Characterization of Selenium Nanoparticle Composites Modified by Astragalus Polysaccharide and the Cytotoxicity Mechanism on Liver Cancer Cells. Molecules.

[21] Tritean N., Dima Ș.O., Trică B., Stoica R., Ghiurea M., Moraru I., Cimpean A., Oancea F., Constantinescu-Aruxandei D. (2023). Selenium-Fortified Kombucha–Pollen Beverage by In Situ Biosynthesized Selenium Nanoparticles with High Biocompatibility and Antioxidant Activity. Antioxidants.

[22] Wang B.W., Baowei W., Guoqing H., Qiaoli W., Bin Y. (2011). Effects of yeast selenium supplementation on the growth performance, meat quality, immunity, and antioxidant capacity of goose. J. Anim. Physiol. Anim. Nutr..

[23] Fan C., Yu B., Chen D.W. (2009). Effects of different sources and levels of selenium on performance, thyroid function and antioxidant status in stressed broiler chickens. Int. J. Poult. Sci..

[24] Ibrahim R.Y.M., Saber A.A., Hammad H.B.I. (2021). The possible role of the seaweed Ulva fasciata on ameliorating hyperthyroidism-associated heart inflammations in a rat model. Environ. Sci. Pollut. Res..

[25] Qiu S.J. (2023). Effects of Nanoselenium-Fucoidan Oligosaccharides on Growth Performance and Intestinal Mechanical Barrier in Heat-Stressed Broilers. Master’s Thesis.

[26] Khajeh Bami M., Afsharmanesh M., Ebrahimnejad H. (2020). Effect of dietary Bacillus coagulans and different forms of zinc on performance, intestinal microbiota, carcass and meat quality of broiler chickens. Probiotics Antimicrob. Proteins.

[27] Yue Y.B., Zou L., Tao J., Yin L., Xie Z.R., Xia Y., Zhang Z.Y., Wang K.H., Zhu M. (2023). Transcriptomics and metabolomics together reveal the underlying mechanism of heroin hepatotoxicity. Toxicology.

[28] Zhang S., Ji O., Zheng L., Lo H.K. (2020). Effect of dietary β-1,3-glucan supplementation and heat stress on growth performance, nutrient digestibility, meat quality, organ weight, ileum microbiota, and immunity in broilers. Poult. Sci..

[29] Tavaniello S., Slawinska A., Prioriello D., Petrecca V., Bertocchi M., Zampiga M., Salvatori G., Maiorano G. (2020). Effect of galactooligosaccharides delivered in ovo on meat quality traits of broiler chickens exposed to heat stress. Poult. Sci..

[30] Wen C., Chen Y., Leng Z., Ding L., Wang T., Zhou Y. (2019). Dietary betaine improves meat quality and oxidative status of broilers under heat stress. J. Sci. Food Agric..

[31] Suliman G.M., Hussein E.O., Al-Owaimer A.N., Alhotan R.A., AlGaradi M.A., Mahdi J.M., Ba-Awadh H.A., Qaid M.M., Swelum A.A.A. (2023). Betaine and nano-emulsified vegetable oil supplementation for improving carcass and meat quality characteristics of broiler chickens under heat stress conditions. Front. Vet. Sci..

[32] Choi J., Kong B., Bowker B.C., Zhuang H., Kim W.K. (2023). Nutritional Strategies to Improve Meat Quality and Composition in the Challenging Conditions of Broiler Production: A Review. Animals.

[33] Zhao Y., Li Z., Wang X., Zhao F., Wang C., Zhang Q., Chen X., Geng Z., Zhang C. (2022). Resveratrol Attenuates Heat Stress-Induced Impairment of Meat Quality in Broilers by Regulating the Nrf2 Signaling Pathway. Animals.

[34] Mir N.A., Rafiq A., Kumar F., Singh V., Shukla V. (2017). Determinants of broiler chicken meat quality and factors affecting them: A review. J. Food Sci. Technol..

[35] Chen S., Liu H., Zhang J., Zhou B., He X., Wang T., Wang C. (2023). Dietary rutin improves breast meat quality in heat-stressed broilers and protects mitochondria from oxidative attack via the AMPK/PINK1–Parkin pathway. J. Sci. Food Agric..

[36] Teyssier J.R., Preynat A., Cozannet P., Briens M., Mauromoustakos A., Greene E.S., Owens C.M., Dridi S., Rochell S.J. (2022). Constant and cyclic chronic heat stress models differentially influence growth performance, carcass traits and meat quality of broilers. Poult. Sci..

[37] Liu Z., Liu Y.S., Xing T., Li J.L., Zhang L., Jiang Y., Gao F. (2022). Transcriptome analysis reveals the mechanism of chronic heat stress on meat quality of broilers. J. Anim. Sci. Biotechnol..

[38] Zhang K., Li X.M., Zhao J.S., Wang Y., Hao X.J., Liu K.D., Liu H.W. (2022). Protective effects of chlorogenic acid on the meat quality of oxidatively stressed broilers revealed by integrated metabolomics and antioxidant analysis. Food Funct..

[39] De Grande A., Ducatelle R., Leleu S., Rapp C., Torres C., Petracci M., De Smet S., Michiels J., Haesebrouck F., Van Immerseel F. (2022). Effects of the dietary zinc source and vitamin E level on live weight and carcass yield and meat quality in male broilers reared under chronic cyclic heat stress conditions in the finisher phase. Front. Physiol..

[40] Mohamed D.A., Sazili A.Q., Teck Chwen L., Samsudin A.A. (2020). Effect of microbiota-selenoprotein on meat selenium content and meat quality of broiler chickens. Animals.

[41] Lochi G.M., Shah M.G., Gandahi J.A., Gadahi J.A., Hadi S.A., Farooq T., Vistro W.A., Rahmani M.M. (2023). Effect of selenium nanoparticles and chitosan on production performance and antioxidant integrity of heat-stressed broiler. Biol. Trace Elem. Res..

[42] Wang C.L., Xing G.Z., Wang L.S., Li S.F., Zhang L.Y., Lu L., Luo X.G., Liao X.D. (2021). Effects of selenium source and level on growth performance, antioxidative ability and meat quality of broilers. J. Integr. Agric..

[43] Xu X., Zhu Y.F., Wei Y., Chen X.F., Li R., Xie J.H., Wang G.G., Ming J.J., Yin H.Q., Xiang J.Q. (2022). Dietary Se-Enriched Cardamine enshiensis Supplementation Alleviates Transport-Stress-Induced Body Weight Loss, Anti-Oxidative Capacity and Meat Quality Impairments of Broilers. Animals.

[44] Khan M.T., Niazi A.S., Arslan M., Azhar M., Asad T., Raziq F., Gondal M.A., Rauf M., Liaqat S., Naz S. (2023). Effects of selenium supplementation on the growth performance, slaughter characteristics, and blood biochemistry of naked neck chicken. Poult. Sci..

[45] Jiang J., Tang X., Xue Y., Lin G., Xiong Y.L. (2017). Dietary linseed oil supplemented with organic selenium improved the fatty acid nutritional profile, muscular selenium deposition, water retention, and tenderness of fresh pork. Meat Sci..

[46] Zhou X., Wang Y. (2011). Influence of dietary nano elemental selenium on growth performance, tissue selenium distribution, meat quality, and glutathione peroxidase activity in Guangxi Yellow chicken. Poult. Sci..

[47] Yang M.X., Lu Z.J., Li F.L., Shi F., Zhan F.B., Zhang Y.L., Zhao L.J., Li Y., Li J., Lin L. (2021). Alginate oligosaccharide improves fat metabolism and antioxidant capacity in the liver of grass carp (*Ctenopharyngodon idellus*). Aquaculture.

[48] Ramanathan R., Kiyimba F., Suman S.P., Mafi G.G. (2023). The potential of metabolomics in meat science: Current applications, future trends, and challenges. J. Proteom..

[49] Shi K., Zhao Q., Shao M.H., Duan Y., Li D.F., Lu Y.Q., Tang Y.F., Feng C.G. (2022). Untargeted Metabolomics Reveals the Effect of Selective Breeding on the Quality of Chicken Meat. Metabolites.

[50] Fang M.X., Hu W., Liu B. (2023). Effects of nano-selenium on cecum microbial community and metabolomics in chickens challenged with Ochratoxin A. Front. Vet. Sci..

[51] Bejaoui B., Sdiri C., Souf I.B., Slimen I.B., Larbi M.B., Koumba S., Martin P., M’Hamdi N. (2023). Physicochemical Properties, Antioxidant Markers, and Meat Quality as Affected by Heat Stress: A Review. Molecules.

[52] Zhang H.Y., Majdeddin M., Degroote J., Liefferinge E.V., Noten N.V., Kerschaver C.V., Vandaele M., Dorigam J.C.D.P., Michiels J. (2023). Effect of supplemental methyl sulfonyl methane on performance, carcass and meat quality and oxidative status in chronic cyclic heat-stressed finishing broilers. Poult. Sci..

[53] Wen C., Liu Y., Ye Y.W., Tao Z.G., Cheng Z.J., Wang T., Zhou Y.M. (2020). Effects of gingerols-rich extract of ginger on growth performance, serum metabolites, meat quality and antioxidant activity of heat-stressed broilers. J. Therm. Biol..

[54] Wang L.W., Su S.F., Zhao J., He X.L., Fu S.Y., Wang B., Wang Y.F., Wang D.Q., Yun N.N., Chen X. (2023). Effects of dietary oat supplementation on carcass traits, muscle metabolites, amino acid profiles, and its association with meat quality of Small-tail Han sheep. Food Chem..

[55] Ma X., Yu M., Liu Z., Deng D., Cui Y., Tian Z., Wang G. (2020). Effect of amino acids and their derivatives on meat quality of finishing pigs. J. Food Sci. Technol..

[56] Zhang Z.Y., Jia G.Q., Zuo J.J., Zhang Y., Lei J., Ren L., Feng D.Y. (2012). Effects of constant and cyclic heat stress on muscle metabolism and meat quality of broiler breast fillet and thigh meat. Poult. Sci..

[57] Gonzalez-Rivas P.A., Chauhan S.S., Ha M., Fegan N., Dunshea F.R., Warner R.D. (2020). Effects of heat stress on animal physiology, metabolism, and meat quality: A review. Meat Sci..

[58] Yin C., Tang S., Liu L., Cao A., Xie J., Zhang H. (2021). Effects of Bile Acids on Growth Performance and Lipid Metabolism during Chronic Heat Stress in Broiler Chickens. Animals.

[59] Wang Y., Liu X., Wang Y., Zhao G., Wen J., Cui H. (2022). Metabolomics-Based Analysis of the Major Taste Contributors of Meat by Comparing Differences in Muscle Tissue between Chickens and Common Livestock Species. Foods.

[60] Tian X.Z., Li J.X., Luo Q.Y., Wang X., Xiao M.M., Zhou D., Lu Q., Chen X. (2022). Effect of supplementation with selenium-yeast on muscle antioxidant activity, meat quality, fatty acids and amino acids in goats. Front. Vet. Sci..

[61] Cruvinel J.M., Groff Urayama P.M., Oura C.Y., de Lima Krenchinski F.K., dos Santos T.S., de Souza B.A., Kadri S.M., Correa C.R., Sartori J.R., Pezzato A.C. (2023). Pequi Oil (*Caryocar brasiliense* Camb.) Attenuates the Adverse Effects of Cyclical Heat Stress and Modulates the Oxidative Stress-Related Genes in Broiler Chickens. Animals.

[62] Hu H., Bai X., Xu K., Zhang C., Chen L. (2021). Effect of phloretin on growth performance, serum biochemical parameters and antioxidant profile in heat-stressed broilers. Poult. Sci..

[63] Tang L.P., Liu Y.L., Zhang J.X., Ding K.N., Lu M.H., He Y.M. (2022). Heat stress in broilers of liver injury effects of heat stress on oxidative stress and autophagy in liver of broilers. Poult. Sci..

[64] Alian H.A., Samy H.M., Ibrahim M.T., Mahmoud M.M. (2020). Nanoselenium effect on growth performance, carcass traits, antioxidant activity, and immune status of broilers. Environ. Sci. Pollut. Res..

[65] Zhang F., Li X., Wei Y. (2023). Selenium and Selenoproteins in Health. Biomolecules.

[66] Jing J.Z., Zeng H.J., Shao Q.J., Tang J.Y., Wang L.Q., Jia G., Liu G.M., Chen X.L., Tian G., Cai J.Y. (2023). Selenomethionine alleviates environmental heat stress induced hepatic lipid accumulation and glycogen infiltration of broilers via maintaining mitochondrial and endoplasmic reticulum homeostasis. Redox Biol..

[67] Cao L., Tang J., Li Q., Xu J., Jia G., Liu G., Chen X., Shang H., Cai J., Zhao H. (2016). Expression of selenoprotein genes is affected by heat stress in IPEC-J2 cells. Biol. Trace Elem. Res..

[68] Abo El-Magd N.F., Barbosa P.O., Nick J., Covalero V., Grignetti G., Bermano G. (2022). Selenium, as selenite, prevents adipogenesis by modulating selenoproteins gene expression and oxidative stress–related genes. Nutrition.

[69] Hu H., Dai S., Li J., Wen A., Bai X. (2020). Glutamine improves heat stress–induced oxidative damage in the broiler thigh muscle by activating the nuclear factor erythroid 2–related 2/Kelch-like ECH-associated protein 1 signaling pathway. Poult. Sci..

[70] Song D.Z., Cheng Y.Z., Li X.X., Wang F.Q., Lu Z.Q., Xiao X., Wang Y.Z. (2017). Biogenic Nanoselenium Particles Effectively Attenuate Oxidative Stress-Induced Intestinal Epithelial Barrier Injury by Activating the Nrf2 Antioxidant Pathway. ACS Appl. Mater. Interfaces.

[71] Zhang Y., Qin S., Song Y., Yuan J., Hu S., Chen M., Li L. (2022). Alginate oligosaccharide alleviated cisplatin-induced kidney oxidative stress via lactobacillus genus-FAHFAs-Nrf2 Axis in mice. Front. Immunol..

[72] Lu J., Zhao Y., Liu M., Lu J., Guan S. (2021). Toward improved human health: Nrf2 plays a critical role in regulating ferroptosis. Food Funct..

[73] Liu J., Huang C., Liu J., Meng C., Gu Q., Du X., Yan M., Yu Y., Liu F., Xia C. (2023). Nrf2 and its dependent autophagy activation cooperatively counteract ferroptosis to alleviate acute liver injury. Pharmacol. Res..

[74] El-Benhawy S.A., Abdelrhman I.G., Sadek N.A., Fahmy E.I., AboGabal A.A., Elmasry H., Saleh S.A., Sakr O.A., Elwany M.N., Rabie M.A.F. (2023). Studying ferroptosis and iron metabolism pre-and post-radiotherapy treatment in breast cancer patients. J. Egypt. Natl. Cancer Inst..

[75] Lin J.H., Yang K.T., Lee W.S., Ting P.C., Luo Y.P., Lin D.J., Wang Y.S., Chang J.C. (2022). Xanthohumol Protects the Rat Myocardium against Ischemia/Reperfusion Injury-Induced Ferroptosis. Oxid. Med. Cell. Longev..

[76] Qiu W., Ye J., Su Y., Zhang X., Pang X., Liao J., Wang R., Zhao C., Zhang H., Hu L. (2023). Co-exposure to environmentally relevant concentrations of cadmium and polystyrene nanoplastics induced oxidative stress, ferroptosis and excessive mitophagy in mice kidney. Environ. Pollut..

[77] Zhu X., Huang N., Ji Y., Sheng X., Huo J., Zhu Y., Huang M., He W., Ma J. (2023). Brusatol induces ferroptosis in oesophageal squamous cell carcinoma by repressing GSH synthesis and increasing the labile iron pool via inhibition of the NRF2 pathway. Biomed. Pharmacother..

[78] Schimanski L.M., Drakesmith H., Merryweather-Clarke A.T., Viprakasit V., Edwards J.P., Sweetland E., Bastin J.M., Cowley D., Chinthammitr Y., Robson K.J. (2005). In vitro functional analysis of human ferroportin (FPN) and hemochromatosis-associated FPN mutations. Blood.

[79] Liu C., Lu J., Yuan T., Xie L., Zhang L. (2023). EPC-exosomal miR-26a-5p improves airway remodeling in COPD by inhibiting ferroptosis of bronchial epithelial cells via PTGS2/PGE2 signaling pathway. Sci. Rep..

[80] Yi T.T., Zhang L.M., Huang X.N. (2023). Glycyrrhizic acid protects against temporal lobe epilepsy in young rats by regulating neuronal ferroptosis through the miR-194-5p/PTGS2 axis. Kaohsiung J. Med. Sci..

[81] Zhao L., Feng Y., Xu Z.J. (2021). Selenium mitigated aflatoxin B1-induced cardiotoxicity with potential regulation of 4 selenoproteins and ferroptosis signaling in chicks. Food Chem. Toxicol..

[82] Jung Y.J., Choi H., Oh E. (2023). Selenium mitigates ferroptosis-mediated dopaminergic cell death by regulating the Nrf2/GPX4 pathway. Neurosci. Lett..

[83] Wu H., Luan Y., Wang H., Zhang P., Liu S., Wang P., Cao Y., Sun H., Wu L. (2022). Selenium inhibits ferroptosis and ameliorates autistic-like behaviors of BTBR mice by regulating the Nrf2/GPx4 pathway. Brain Res. Bull..

