# Supplementary material for: Biogenic Selenium Nanoparticles Synthesized Using Alginate Oligosaccharides Attenuate Heat Stress-Induced Impairment of Breast Meat Quality via Regulating Oxidative Stress, Metabolome and Ferroptosis in Broilers
Source: Antioxidants (Basel). 2023 Nov 22;12(12):2032. doi: 10.3390/antiox12122032 (PMC10740886; doi:10.3390/antiox12122032)
Supplement: Supplementary file 1 [file antioxidants-12-02032-s001.zip › Table S1.pdf]

Table S1. Basal diet composition and ingredients（22-42d）.

Item	Contents (%)
Ingredients	
Corn	55.00
Soybean meal	34.82
Wheat bran	2.00
Soybean oil	5.00
Limestone	0.50
CaHPO4	1.60
NaCl	0.30
DL-Methionine	0.18
L-Lysine (50%)	0.10
Vitamin Premix [1]	0.20
Mineral premix [2]	0.30
total	100
Nutrient levels	
ME (MJ/kg)	12.82
Crude protein (%)	19.92
Ca (%)	0.93
P (%)	0.44
Lys (%)	1.30
Met (%)	0.45
Total Met + Cystine (%)	0.72
Se (%) [3]	Measured value

[1] The premix provides per kg of feed: vitamin A 9000 IU, vitamin D3 3240 IU, vitamin E 6 IU, vitamin K3 0.75 mg, vitamin B1 1.5 mg, vitamin B2 4.5 mg, vitamin B6 1.5 mg, niacin 9 mg, folic acid 0.45 mg, pantothenic acid 9 mg, choline 1000 mg.

[2] Fe 80 mg, Cu 8 mg, Mn 60 mg, Zn 40 mg, I 0.35 mg. Mn 60 mg, Zn 40 mg, Se 0.15 mg, and I 0.35 mg.

[3] Selenium is measured value, selenium content of the basal diet was 0.282 mg/kg, selenium content of the diet in the SeNPs-AOS addition group was 0.696 mg/kg, and the rest are calculated values.
