# Supplementary material for: Biogenic Selenium Nanoparticles Synthesized Using Alginate Oligosaccharides Attenuate Heat Stress-Induced Impairment of Breast Meat Quality via Regulating Oxidative Stress, Metabolome and Ferroptosis in Broilers
Source: Antioxidants (Basel). 2023 Nov 22;12(12):2032. doi: 10.3390/antiox12122032 (PMC10740886; doi:10.3390/antiox12122032)
Supplement: Supplementary file 1 [file antioxidants-12-02032-s001.zip › Table S2.pdf]

Table S2. Primers used for quantitative real-time PCR.

Target Genes	Primer	Primer Sequence (5'→3')	Accession No.
CAT	Forward	CTCCTGGCTTACTCGTTGGT	NM_001145490
	Reverse	CTCATTGTTATCTGTCTCCTCTGTT	
SOD1	Forward	AGGGAGGAGTGGCAGAAGT	NM_205064.1
	Reverse	GCTAAACGAGGTCCAGCAT	
SOD2	Forward	TCCTGACCTGCCTTACGACTATGG	NM_204211.1
	Reverse	GCGACACCTGAGCTGTAACATCAC	
GSTT1	Forward	CATGCTAACATCCGGGCTAA	NM_205365.1
	Reverse	AAATTGCTTCAGGGAAGTGG	
GPX1	Forward	CAAAGTGCTGCTGGTGGTCAAC	NM_001277853.2
	Reverse	TTGGTGGCGTTCTCCTGGTG	
GPX3	Forward	TGGCAGAGGAGTTCGGCAAC	NM_001163232.2
	Reverse	CGTTCTTGACAGTGGCGATGTT	
HO-1	Forward	GCCTACACCCGCTATTTGG	NM_205344.1
	Reverse	TCTCAAGGGCATTCATTCG	
Keap1	Forward	ACTTCGCTGAGGTCTCCAAG	NM_012289.4
	Reverse	CAGTCGTACTGCACCCAGTT	
MafF	Forward	CGACGACGGACGCTGAAGAA	NM_204757.2
	Reverse	GTACTTGCCACGGAGAGTGTCAA	
MafG	Forward	ACGCTGAAGAACCGAGGCTAC	NM_001079489.1
	Reverse	GTTCTGGCGAAGTTCTGGAGTG	
MafK	Forward	GCAGCAAGAGGTGGAGAAGC	NM_204756.2
	Reverse	ACGGCACGGAACTGGATGA	
SELENOS	Forward	CGTCGCCATCTATCTCATCGT	NM_001024734.3
	Reverse	GCTTCTTGTCTTCTTACCACCAT	
SELENOW	Forward	CAGGAGGTGACGGGATGGTT	NM_001166327.2
	Reverse	TACGGGAGGGCAGCTTGGAT	
SELENOT	Forward	GGCACATAGCATCCTTCCTG	NM_001006557.4
	Reverse	CCGTTGACATACACTGGTTCT	
SELENOK	Forward	CCAGAGCATTATTCAACCAGACCT	NM_001025441.2
	Reverse	CCTCATCCACCTCCAGCCATT	
GPX4	Forward	ACCCGCTGTGGAAGTGGATGAAG	NM_001039848.4
	Reverse	TCACCACGCAGCCGTTCTTGT	
FTH1	Forward	GAATGTGAACCAGTCGCTGTTAGA	NM_205086.2
	Reverse	AGGTACTCTGCCATGCCATACTT	
Fpn1	Forward	CCACAGCGATCACAATTCAGAGG	NM_001012913.2
	Reverse	CGACATCAGGTTCCAGCCAGAA	
TERT	Forward	TTCCTCGCTCCTCCCTCAGT	NM_001031007.2
	Reverse	CGGCATTTGTTATGGCTTGAACC	
Nrf2	Forward	TGTGTGTGATTCAACCCGACT	NM_205117.1

	Reverse	TTAATGGAAGCCGCACCACT	
SLC7A11	Forward	CTGTCGTGACGGTGCCTAA	XM_426289.7
	Reverse	CCAATGATAGTGCCAATGATGATG	
PTGS2	Forward	TGGTGAGACTCTGGAGAGGCAACT	NM_001167718.2
	Reverse	GCCAAACACCTCCTGCCCAACA	
β-actin	Forward	GTGATGGACTCTGGTGATGGTGTT	NM_205518.1
	Reverse	TCTCGGCTGTGGTGGTGAAG	
